# Home-based cooking intervention with a smartphone app to improve eating behaviors in children aged 7–9 years: a feasibility study

**DOI:** 10.1007/s44155-023-00042-4

**Published:** 2023-06-01

**Authors:** Joyce Haddad, Maria F. Vasiloglou, Franziska Scheidegger-Balmer, Ulrich Fiedler, Klazine van der Horst

**Affiliations:** 1 Bern University of Applied Sciences, School of Health Professions, Nutrition and Dietetics, Murtenstrasse 10, 3008 Bern, Switzerland; 2 AI in Health and Nutrition Laboratory, ARTORG Center for Biomedical Engineering Research, University of Bern, Bern, Switzerland; 3 Institute ICE, School of Engineering and Computer Science, Bern University of Applied Sciences, Biel/Bienne, Switzerland

**Keywords:** Food acceptance, Liking, Children, Cooking, Meal preparation, Vegetable intake, Food intake

## Abstract

**Objective:**

To develop and evaluate the feasibility of a mobile application in Swiss households and assess its impact on dietary behavior and food acceptability between children who cooked with limited parental support (intervention group) with children who were not involved in cooking (control group).

**Methods:**

A ten-week randomized controlled trial was conducted online in 2020. Parents were given access to a mobile-app with ten recipes. Each recipe emphasized one of two generally disliked foods (Brussels sprouts or whole-meal pasta). Parents photographed and weighed the food components from the child’s plate and reported whether their child liked the meal and target food. The main outcome measures were target food intake and acceptability analyzed through descriptive analysis for pre-post changes.

**Results:**

Of 24 parents who completed the baseline questionnaires, 18 parents and their children (median age: 8 years) completed the evaluation phase. Mean child baseline Brussel sprouts and whole-meal pasta intakes were 19.0 ± 24.2 g and 86.0 ± 69.7 g per meal, respectively. No meaningful differences in intake were found post-intervention or between groups. More children reported a neutral or positive liking towards the whole-meal pasta in the intervention group compared to those in the control group. No change was found for liking of Brussel sprouts.

**Conclusions for practice:**

The intervention was found to be feasible however more studies on larger samples are needed to validate feasibility. Integrating digital interventions in the home and promoting meal preparation may improve child reported acceptance of some healthy foods. Using such technology may save time for parents and engage families in consuming healthier meals.

## Significance

Research on the exploration and development of interventions in real-life home settings with extended periods of follow‐up is scarce. The effects of children’s cooking involvement on intake of core food groups, other than vegetable and fruit, are not fully known.

This was the first Swiss study to test the feasibility of an innovative pilot intervention for a home-based cooking program. Despite the lack of significant results, this pilot study can be used as a base for future trials focusing children’s dietary behavior improvement, and pave the way to identifying innovative methods to save time for parents and engage families to cook and consume healthier meals.

## Introduction

In Switzerland, children aged 5–12 years are not adhering to national dietary guidelines for six food groups, especially as 46% and 5% are meeting fruit and vegetable recommendations, respectively [1]. Early promotion of healthy dietary behavior is crucial because eating habits established in childhood usually continue into adulthood [2].

The family environment plays a crucial role in establishing children’s healthy dietary behaviors [3] by parental healthy behavior modeling, the availability and accessibility of a variety of healthy food [4–6], and early exposure to tastes and flavors [7]. A systematic review and meta-analysis of parent-targeted home-based interventions showed that early taste exposure interventions can lead to a significant increase in vegetable intake [8]. Involvement in meal preparation can also be an avenue for promoting food preferences and healthy food intake [9–13].

Several reviews of children’s cooking interventions recommend future research on school-based cooking initiatives [14–16]. However, as parents are the ‘gatekeepers’ of the home and have a central role in creating the environments which shape their children’s diet, home-based interventions should be prioritized for improving children’s dietary behaviors [8]. Often, nutrition education programs in school settings include multiple components such as tasting lessons, gardening activities and meal preparation activities [17]. Some findings suggest that that school-based interventions are not as effective as the home environment for promoting fruit and vegetable intake, highlighting the importance for focusing on families for promoting healthy eating behavior [3]. Shifting the intervention focus from only a school-setting to incorporate home-settings is warranted. The factors that have been associated with improved fruit and vegetable intake in school settings include the use of experiential learning strategies, i.e., involving children in hands-on activities such as gardening and meal preparation [6, 18, 19]. Visual, practical, and sensory learning techniques have also previously resulted in positive parent–child interaction and bonding [20]. Such experiential learning can be implemented in home-based interventions.

Parent and child cooking interventions have mostly taken place through workshops or community-based interventions [9, 10, 21, 22]. However, in a rapidly changing environment, time scarcity is often a barrier to improving healthy behaviors [23]. As a solution, digital tools such as mobile applications can be used conveniently in different research settings and can have farther reach than traditional methods [24, 25]. Additionally, children and parents report a preference towards e- and m-Health interventions compared to paper-based approaches [26]. Children and parents enjoy the practical, engaging and tailoring advantages of digital technology, as well as the flexibility in the location and the ability for both parents/caregivers to participate [27]. Although there is an abundance of nutrition-related apps, their validity and reliability in scientific trials or as intervention tools can be suboptimal since they may not always be evidence based [28]. High quality interventions are needed to test the feasibility of the apps and other digital technologies [29].

Despite the large number of published digital interventions [30, 31], research in the area of eating behaviors, especially with a focus on children, is scarce [31]. Furthermore, some important research gaps need to be addressed. For example, the exploration and development of interventions in real-life home settings with extended periods of follow‐up are still needed. Additionally, research on the effects of cooking involvement have been focused on vegetable and fruit consumption. Since children are not meeting the recommendation of many other food groups [1], the effect of cooking involvement on a variety of foods, such as wholegrains and pulses, should also be prioritized [32, 33].

Therefore, this study aimed to develop and evaluate the feasibility of an innovative mobile application in German-speaking Swiss households, and assess its impact on dietary behavior and food liking outcomes of two target foods, comparing children who cook with little parental support with those who are not involved in cooking.

## Methods

This feasibility study was designed as a randomized controlled trial (RCT) over a 10-week period. The RCT consisted of 3 phases: (i) the content development of the intervention (recipes, selection of target foods) and the smartphone application (with the research interphase, (ii) the intervention implementation and (iii) the evaluation phase. The study was declared exempt from a full ethical review by the Cantonal Ethics Committee, Bern, Switzerland (BASEC-Nr: Req-2020-00127).

### Intervention: content and application development

- Procedure for choosing the target food-items and recipes creation:

Whole-meal pasta and Brussel sprouts were used as the two target food groups for this intervention. In order to choose the target foods, a small convenience sample of parents (n = 40) in the Bern University of Applied Sciences’ Nutrition and Dietetics research department, were asked which foods were disliked by their children. A list of the following ‘unpopular’ food items were reported by the parents: whole-meal pasta and rice, butternut (pumpkin), celery, radish, Romanesco, cauliflower, radish, eggplant, Brussels sprouts, fish and lentils. The parents reported that their children much preferred refined carbohydrate options and were averse to trying options made with whole-meal flour or wholegrain, such as whole-meal pasta. The parents also reported that Brussel sprouts were the least liked vegetable by their children. To validate these subjective opinions, a literature search was conducted. Whole-meal pasta was chosen as one of the target food for this study [34, 35] because it is known that wholegrain products are less preferred by consumers than their refined-flour counterparts. In children, intakes of whole grains compared to refined grains are significantly less accepted [36, 37]. Brussel sprouts are known to be unpopular amongst children due to their bitter flavor.

Seasonal recipes were created, prepared, and evaluated to rework difficult steps so that they were all suitable for children to make [38]. The recipes did not differ in cooking methods or ingredients between control and intervention group. The recipes were photographed to create images for the application.

- The information technology (IT) procedure of the app creation

The system for the study pertains to the *Kids Cooking@Home* [39] mobile application and a hosted backend. Both were jointly developed using an agile process. The functionality of this system refers to:

1. Preparing by administrators (nutrition team) in the backend: Creating various recipes, assigning participants to intervention and control group, allocating the order and duration of accessibility of the recipes in the mobile application.
2. Implementing by participants using the mobile application: Working through recipes, filling-in questionnaires, and in the background, uploading acquired data to the backend.
3. Evaluating the collected data by administrators (nutrition team) in the backend.

**Fig. 1:**
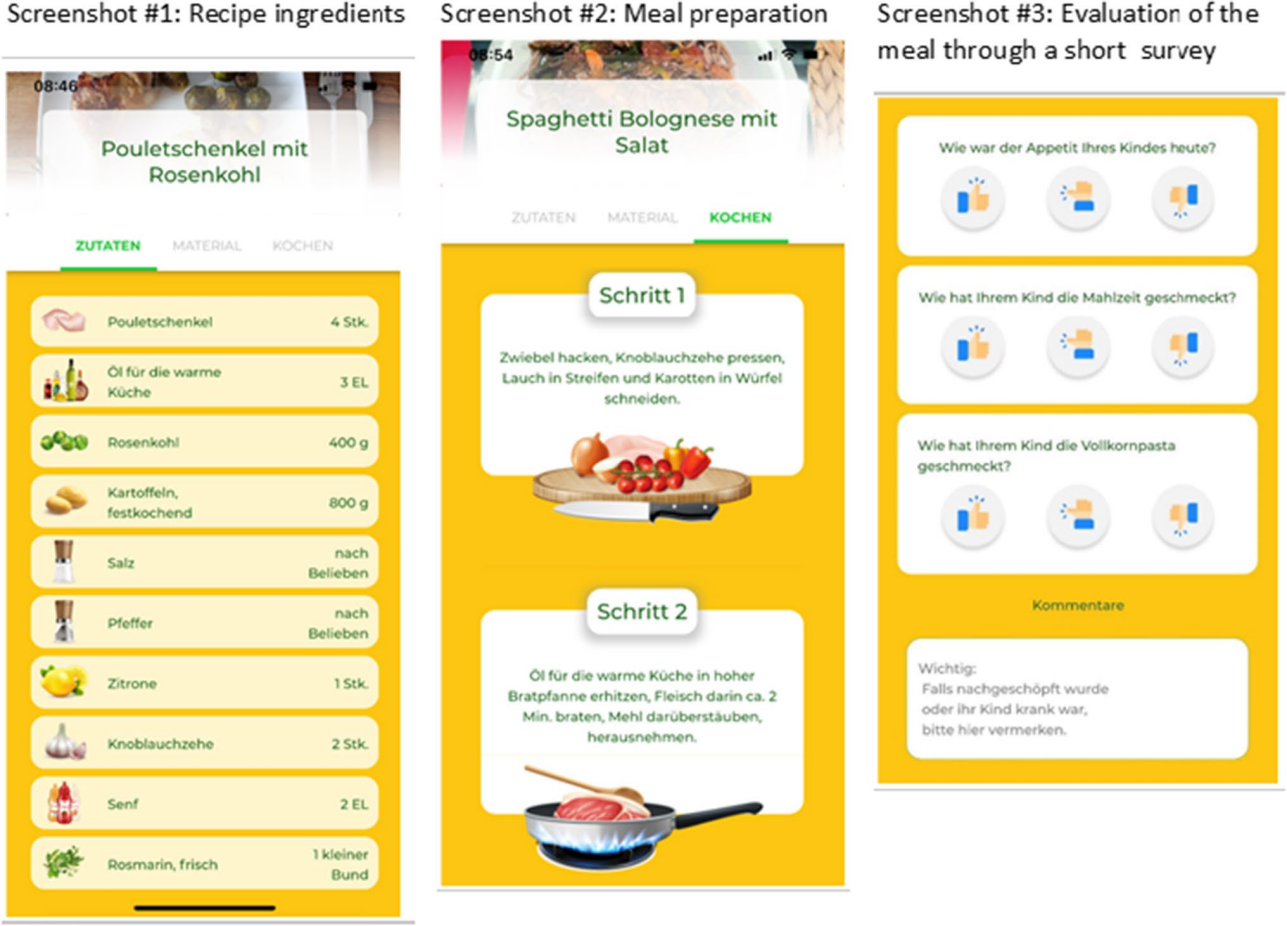
Sample screenshots of the *Kids Cooking@Home* app, depicting the list of required ingredients, the preparation process, and the evaluation of liking of the meal with a short survey

Screenshots of the mobile application are depicted in Fig. 1.

The backend was hosted by Infomaniak [40], the largest web hosting provider in Switzerland. Employed technologies include Google Flutter [41] in the frontend and Java Spring [42]/Spring Boot [43] with a MySQL [44] database in the backend. Throughout the project, special attention was given to data privacy. All data transfers were encrypted and all connections were authenticated.

## Participants

### Recruitment

Children and parents from German-speaking Switzerland were recruited between July and October 2020. The Bern University of Applied Sciences (BFH) Facebook page was used to advertise the study. Interested parents filled out an online questionnaire and were asked about their sociodemographic data (e.g. age and sex of their child), whether their child had special diet, their child’s liking of Brussels sprouts and whole-meal pasta, the child’s eating behavior with respect to foods in general and unfamiliar foods, whether the child cooks with their parents, current disease/allergy/intolerance which affects or restricts food choices, and whether the parent was interested in taking part in a 10-week cooking program, and contact information. The research team contacted all interested parents by telephone to further check their eligibility criteria and to inform the potential participants the study’s details. During the phone call, additional basic demographic characteristics and personal data were gathered that were needed for the personalization and access to the application i.e., name/surname, age, sex, highest education completed, email address and phone number.

Interested parents received a cover letter with general information about the study, a consent form to complete, sign, and send back to the research team. After receiving informed consent, participants were randomly assigned to one of the two intervention groups. Participants received a detailed description of the app installation, a study description and access to a support mail address and telephone number.

### Inclusion and exclusion criteria

Parent and child participants were included if they were German-speaking Swiss residents. Children were included if their age was between 7 and 9 years old, if they were not independently cooking for the family more than once per month and if they were not following a special or restrictive diet. Children were only included if they disliked Brussels sprouts and whole-meal pasta at baseline. Parents were eligible if they reported being able to participate in the intervention over a 10-week period and had a smartphone with Android version 7.0 or iOS version 8.0 to access the mobile-app.

### Intervention implementation

**Fig. 2:**
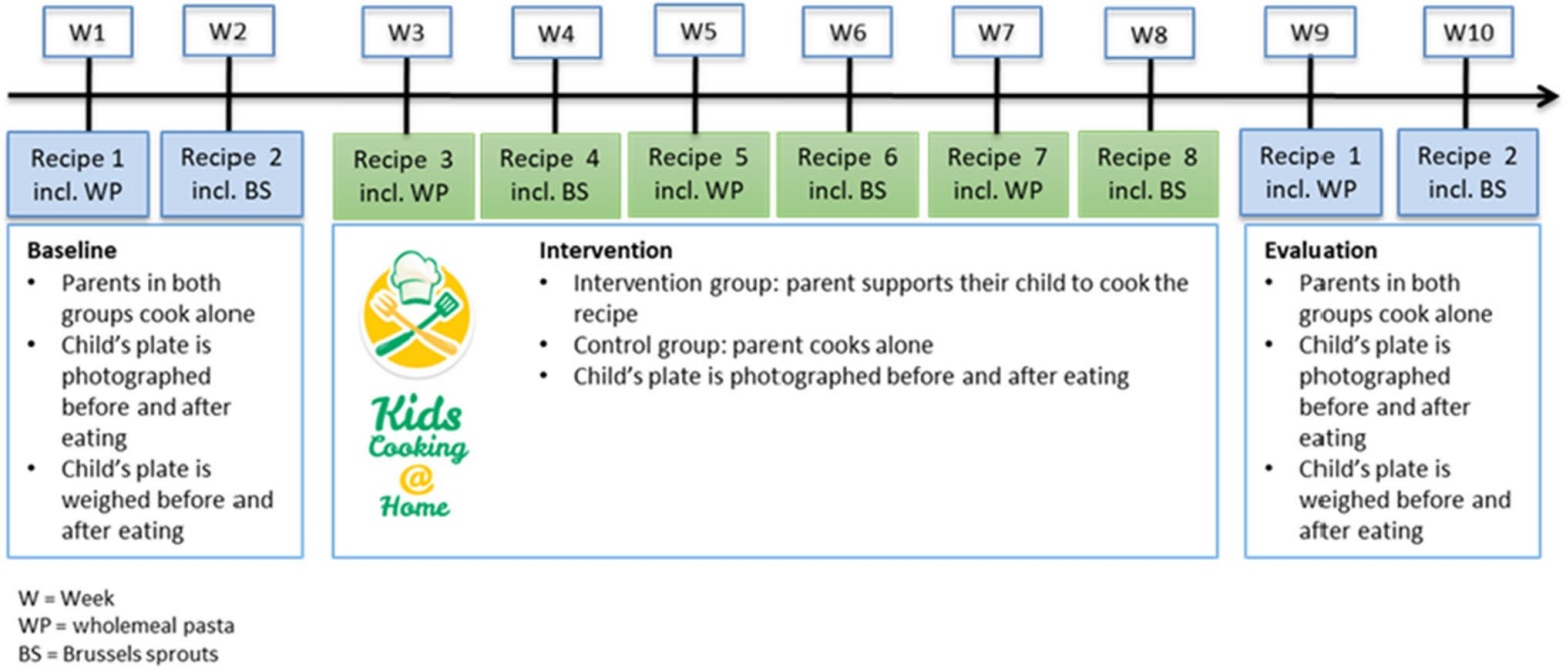
Graphical overview of the 10-weed randomized study

At baseline, parents from both intervention and control groups were required to cook the first two recipes in weeks 1 and 2 alone (Fig. 2). The recipe names, which were available via the application, are found in Table 1. The target ingredient in the recipes was either whole-meal pasta or Brussel sprouts. Each week, a new recipe included one target ingredient in alternating order. After following the recipe and preparing the meal, parents were asked to photograph and weigh the food components (vegetable, meat, carbohydrates) from the child’s plate before and after consumption (in weeks 1, 2, 9 and 10 only). The pictures of the plate, as well as the weight of the food components could be entered in the application before consumption and after consumption in grams. In weeks 3 to 8, parents in the intervention group were asked to only provide minimal support while their child took control of the recipe preparation. Parents were asked to support with the activities less suitable for children, like draining cooked pasta in the sink. In weeks 3–8 parents were not asked to weigh the food on the plates of the children to have a more natural eating setting. Instead, intake could be estimated through the before and after pictures of the plates that were uploaded through the application at baseline and evaluation. Parents in the control group were asked to cook the recipes alone and were following similar instructions. In weeks 9 and 10, the evaluation measurement was conducted by asking all parents to prepare the baseline recipes from weeks 1 and 2 (same recipes) alone and to weigh the foods on the plate before and after eating. The meals that had to be weighed were: (1) whole-meal pasta with pesto, meatballs and grilled tomato, and (2) chicken thighs with Brussels sprouts. These meals were especially provided at baseline and evaluation for ease of separating the target foods for weighing, and to enable a comparison of the child’s food intake between baseline and the end of the intervention.

**Table 1:** Overview of the recipes provided in the *Kids Cooking@Home* app for each intervention group from weeks 1 to 19, and the targeted food for each recipe

	Week of intervention	Recipe provided	Target food	Intervention
Baseline	Week 1	Whole-meal pasta with pesto, meatballs and grilled tomato	Whole-meal pasta	Parents in both groups cook alone
	Week 2	Chicken thighs with Brussels sprouts	Brussel sprouts	
Intervention	Week 3	Spaghetti bolognese with salad	Whole-meal pasta	Intervention group: parent supports their child to cook Control group: parent cooks alone
	Week 4	Noodle soup with Brussels sprouts	Brussel sprouts	
	Week 5	Pasta with peas and ham	Whole-meal pasta	
	Week 6	Toast with egg and Brussels sprouts	Brussel sprouts	
	Week 7	Chicken Sesame Pasta	Whole-meal pasta	
	Week 8	Brussels sprouts and chestnut pasta	Brussel sprouts	
Evaluation	Week 9	Whole-meal pasta with pesto, meatballs and grilled tomato	Whole-meal pasta	Parents in both groups cook alone
	Week 10	Chicken thighs with Brussels sprouts	Brussel sprouts	

After consumption of the recipe was cooked on that day, parents were asked to report whether their child liked the meal; whether their child liked the target food item (i.e., Brussels sprouts or whole-meal pasta) and how the appetite of the child was on that day. This short survey was provided within the application via a 3-point Likert scale: “no” was depicted as a thumbs down/red color, “neither no or yes” was depicted as a neutral thumb / amber color, and “yes” as thumbs up/green color (screenshot #3 in Fig. 1). After the final week of the intervention, qualitative data was collected from four families via telephone interviews, asking questions about their participation in the study in terms of time required and cooking together with their child, what they particularly enjoyed from the experience, what were the challenges they encountered, the comprehension of information and the usability of the app, and what were the positives and negatives related to the recipes in the app. Participants also sent spontaneous feedback by email. An incentive of gaining a children’s cookbook was offered to participants for completing the 10-week study.

## Statistical methods

Descriptive statistics were calculated, means and standard deviations were reported for food consumption data, frequency, and media statistics were reported for participant characteristics and food liking data. No sample size calculation was conducted for this feasibility study, however, we aimed to recruit 30 participants in total. Due to the small sample size in this feasibility study, statistical significance tests were not performed. Data were analyzed using Microsoft Excel and SPSS (Version 28 SPSS Inc., Chicago, Ill., USA).

## Results

### Participants

**Fig. 3:**
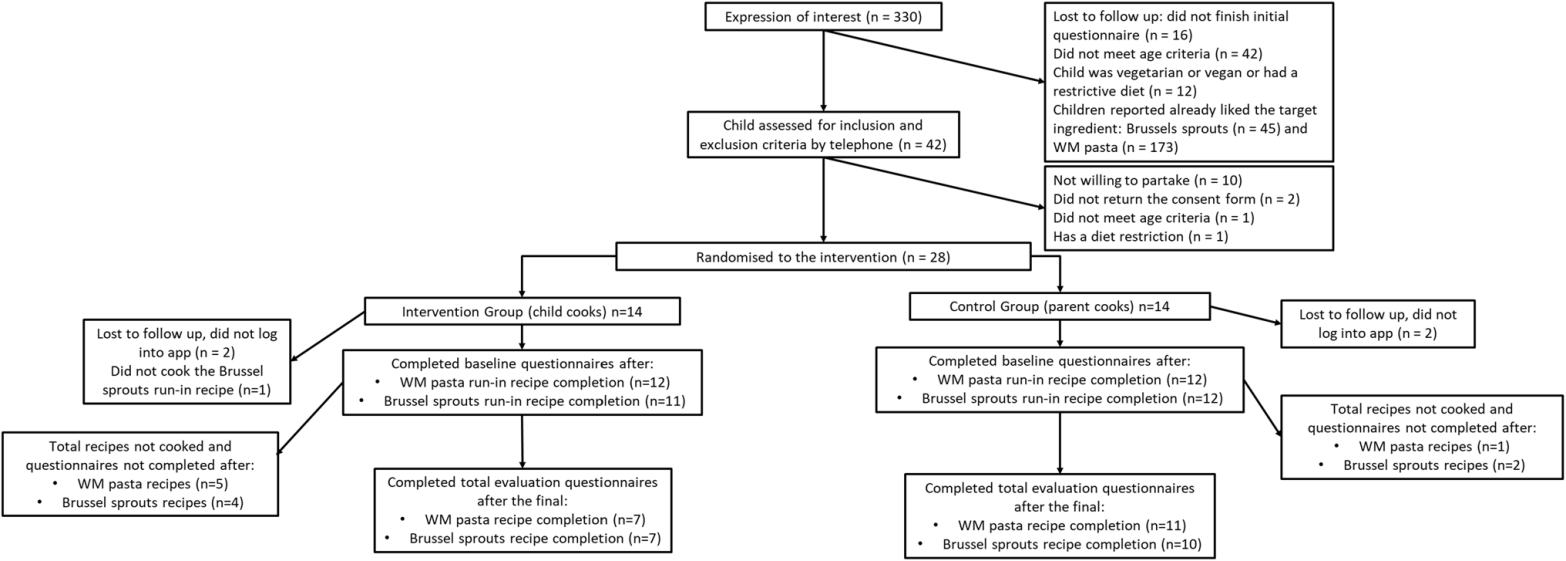
Flowchart of the study participation and reasons for non-completion. WM: whole-meal

A flowchart of participant recruitment is presented in Fig. 3. After the study announcement, 330 parents expressed interest by navigating to the online questionnaire, however 288 were lost to follow up or did not meet study criteria. The contact details of 42 participants who filled out the questionnaire and fulfilled the online inclusion and exclusion criteria of the study were invited for a telephone interview. Of the 42 participants, 28 were willing to partake, met the inclusion and exclusion criteria and returned the consent forms. The participants (n = 28) were randomly allocated to either the intervention or control group. From the intervention group (n = 14), two participants were lost to follow up and did not log in to the application, and one did not cook the run-in recipe. While from the control group (n = 14), two participants were lost to follow up. After the baseline recipes were cooked, a total of seven participants cooked the whole-meal pasta recipes, and seven cooked the Brussel sprouts recipes in the intervention group. In the control group, a total of 11 and 10 whole-meal pasta and Brussels sprout recipes, respectively, were cooked (Fig. 3). This resulted in seven participants completing the study in the intervention group, and 11 in the control group.

The characteristics of the participants who started and completed the study (both parents and children) are presented in Table 2. There were no visually significant differences in the age of parents and children between the intervention and control groups. Of note, one male did complete the evaluation questionnaire on behalf of his female partner, explaining why there is one male at evaluation but not at baseline. Of those that completed the intervention, most parents were female (n = 17) with a university undergraduate degree (n = 10). For the children, the median age was 8 years old, with an equal number of nine female and nine male.

**Table 2:** Parent and child age, sex and education characteristics by intervention group

		Baseline		Evaluation	
		Intervention group (n = 12)	Control group (n = 12)	Intervention group (n = 7)	Control group (n = 11)
Parents’ characteristics					
	Age (median, range)	42 (34–46)	39 (30–50)	41 (37–44)	40 (30–50)
Sex (n)	Female	12	12	6	11
	Male	0	0	1	0
Education (n)	Secondary school	2	6	0	6
	University student	0	1	0	1
	University undergraduate	9	5	6	4
	University postgraduate	1	0	1	0
Children’s characteristics					
	Age (median, range)	8 (7–9)	8 (7–9)	8 (7–9)	8 (7–9)
Sex (n)	Female	7	5	4	5
	Male	5	7	3	6

Data shown for the initial (baseline) sample that started the study, and the final (evaluation) sample that cooked all recipes at the end of the study

### Food intake

Using descriptive analysis, no clear pattern was observed when looking at the mean intake of the target food items (i.e., whole-meal pasta and Brussels sprouts). However, whole-meal pasta intake increased in the intervention group and decreased in the control group. Of note, whole-meal pasta consumption was high at baseline for the control group. In relation to the intake of Brussels sprouts, no changes were observed (Table 3).

**Table 3:** Mean intake of the target food (in grams) at baseline and evaluation

	Baseline target food intake in grams (SD)	n	Evaluation target food intake in grams (SD)	n
Whole-meal pasta recipe and questionnaire completers				
Total group	86 (69.7)	24	86 (62.8)	18
Intervention group	66 (56.1)	12	88 (62.6)	7
Control group	106 (78.4)	12	84 (64.4)	11
Brussels sprouts recipe and questionnaire completers				
Total group	19 (24.2)	23	19 (23.9)	17
Intervention group	20 (23.3)	11	17 (25.5)	7
Control group	19 (26.1)	12	20 (24.1)	10

Data shown by intervention group and for the overall sample that completed the questionnaires at baseline (n = 24) and at evaluation (n = 18)

### Food liking

**Fig. 4:**
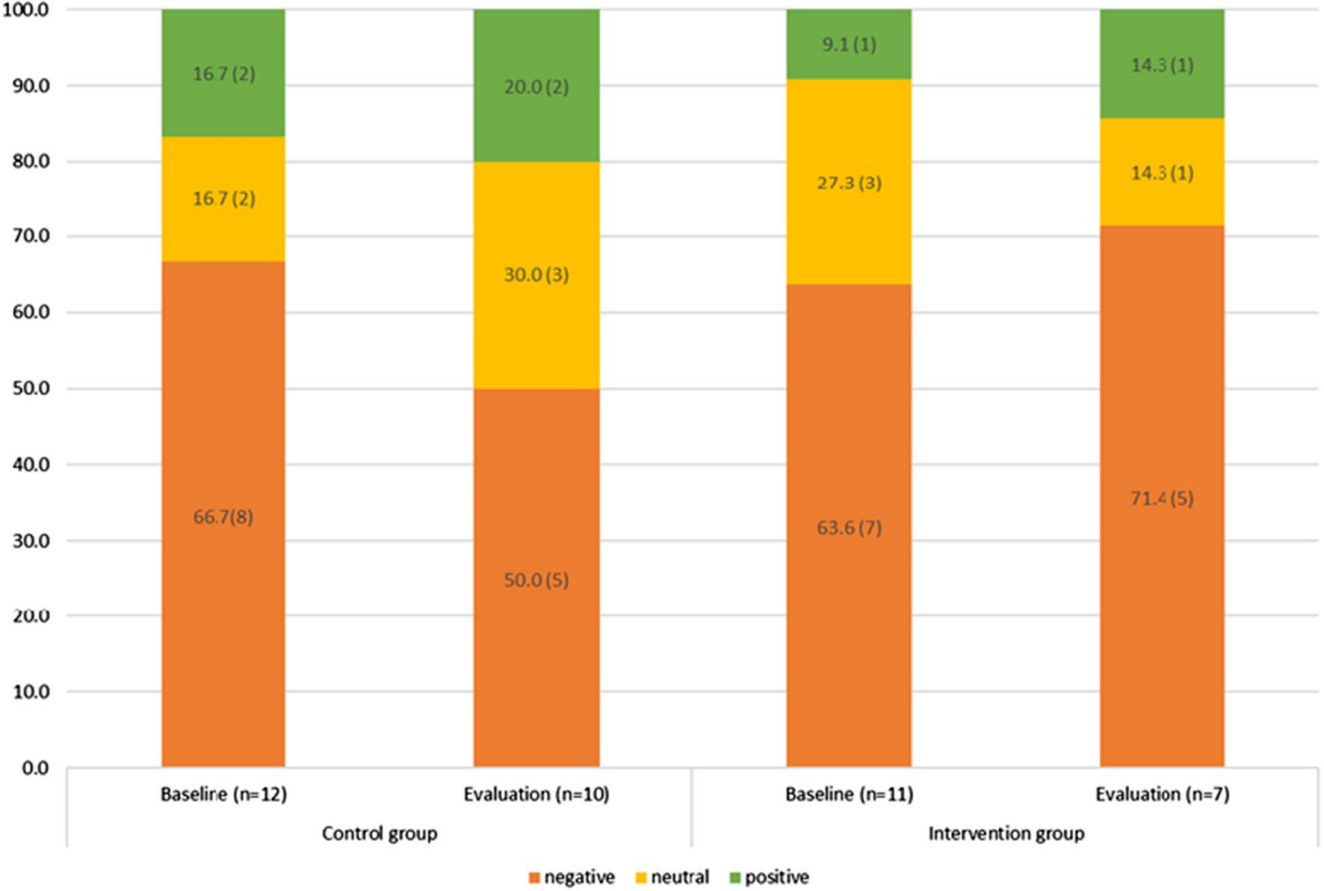
Percentage (n) of participants who reported negative, neutral or positive liking towards Brussels sprouts, by intervention and control group at baseline and evaluation. Data shown for all participants who provided the responses

There was no change in the percentage of children who reported liking the target foods and a slight decrease in those who rated them as neutral. In the control group, a lower percentage of children rated Brussel sprouts liking negatively at evaluation compared to baseline, while a higher percentage of children rated them positively in the intervention group, as per Fig. 4.

**Fig. 5:**
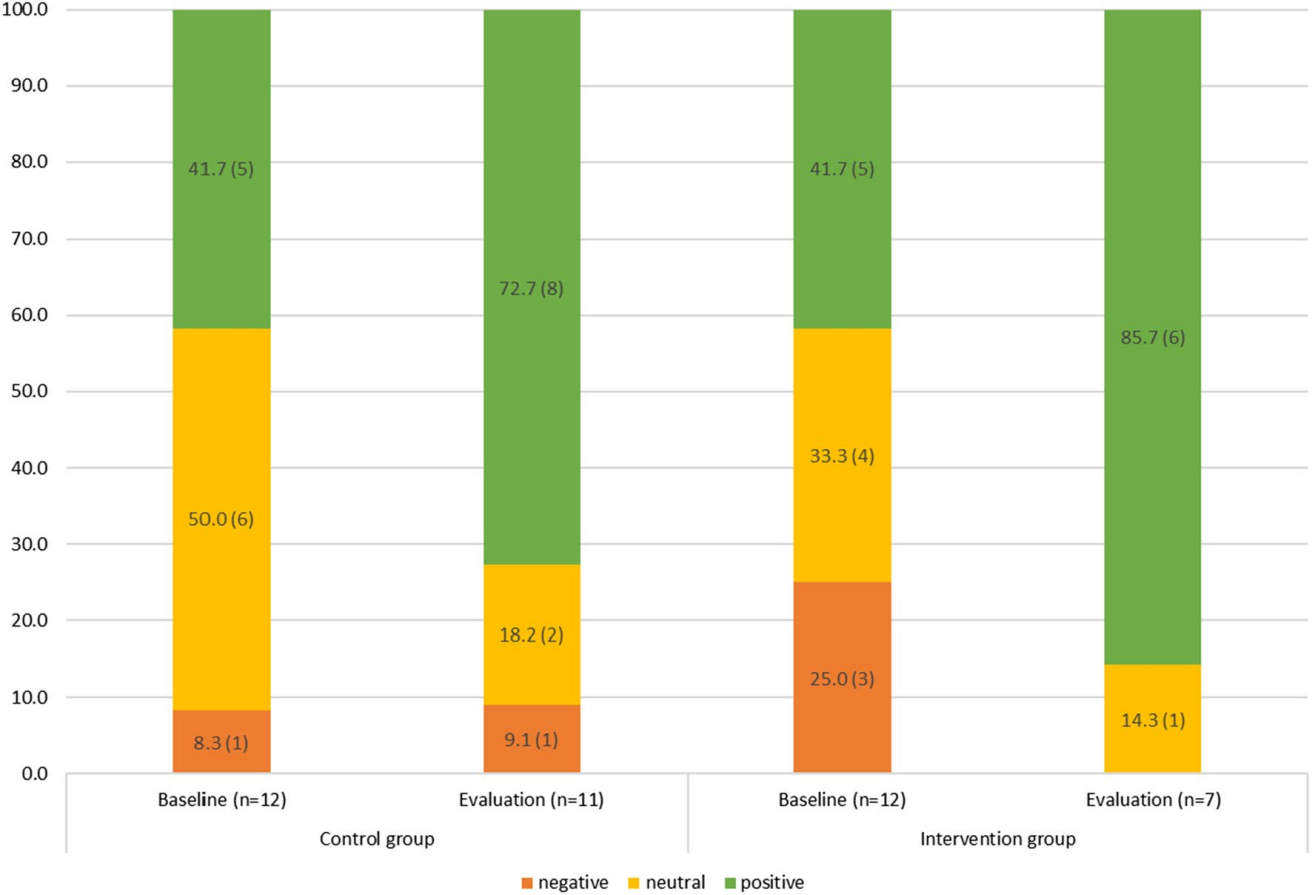
Percentage (n) of participants who reported negative, neutral or positive liking towards whole-meal pasta, by intervention and control group at baseline and evaluation. Data shown for all participants who provided the responses

For the whole meal pasta liking, a higher percentage of children rated the food more positively at evaluation compared to baseline in the control and intervention groups. There were no negative ratings for whole meal pasta at evaluation for the intervention group, as shown in Fig. 5.

No significantly different observations were seen when the data for only the participants who finished the intervention were assessed and compared between control and intervention groups.

### Participants’ feedback

Participant feedback indicated a positive overall reaction to the app. The participants reported that the images were easy to understand, and the support offered through the app was appreciated. there were reports that the new foods were integrated in the family meals after the study conclusion due to the learnt recipes’ time saving and ease. In the intervention group, parents reported that the children appreciated eating whole-meal pasta after the study. The cooking was fun for the children and the whole family appreciated to plan a specific time to cook together, recognizing it as quality time. Children expressed their pride to participate in the study to friends and family. In addition, the parents in the control group noticed a positive impact on the eating behavior of the child, who were more open to trying the new foods and which positively impacted family mealtime.

#### Feedback on aspects that can be improved

Children perceived the study duration as long and missed their usual dishes while younger siblings would also have liked to participate in the cooking procedure. As the recipes were only required to be cooked once a week, a further explanation for this feedback could not be answered by the participants. Parents reported that it was sometimes a challenge to cook with the child due to the need for organization and motivation, and it was more difficult to motivate the child for the Brussel sprouts recipes which were not so popular among the children. In the control group it was difficult to choose the right words when communicating with the child and to know how much he/she should know about the study.

Participants reported partially forgetting or having trouble taking photos of the dish before and/or after the meal and answering the three questions after each meal. They also reported that weighing the meal components before consumption resulted in the food getting cold. Some parents reported that they would have preferred to have the application available on more devices such as tablets, to have an indication of the expected preparation time of the recipes and reminders before the tasks’ due date.

## Discussion

This study aimed to evaluate the feasibility of a mobile app that promoted home-based cooking, and assess its impact on dietary behavior and food acceptability between children who cooked with limited parental support with children who were not involved in cooking. Eighteen parents and their children finalized the randomized controlled study; results showed no difference in vegetable and whole-meal pasta intake before and after the intervention or between intervention and control group. However, there was a slight increase in the number of children in the intervention group who reported a positive liking towards whole-meal pasta post-intervention. Although this study showed limited intervention effect, the repeated exposure of the pasta may have resulted in increased acceptance of the foods and this is in line with many studies showing this effect [45]. Furthermore, allowing children to participate in cooking can have promising potential for improving their dietary behavior. For example, a cross-sectional survey among 305 Swiss parents of 6–12-year-old children showed clear positive associations between the children's cooking enjoyment (e.g., “my child loves to cook”, “my child likes to try new recipes”) and eating enjoyment, and the survey also revealed an inverse association with picky eating behaviors [46]. The limited difference between liking of Brussel sprouts at baseline and evaluation may be explained by this vegetable being very unpopular amongst children, which was portrayed by the feedback received, that the children were demotivated to cook when it came to the Brussel sprout recipes. Nonetheless, it was seen in that less families in the intervention group finished the study. This could be explained by children and parents finding it difficult to cook a meal weekly. This is supported by the feedback of parents who reported cooking with the child to be challenging due to the extra need for organization and motivation. In the future, more flexibility in the intervention could be introduced, such as asking families to cook a number of recipes in a time frame of 10 weeks, with their choice of how often they do it.

Global data has consistently shown similar results. In the US, a study showed that adolescents who were engaged in more frequent dinner preparation at home were more likely to have healthier food intake than those who were not involved [12]. For example, preparation of food seven times in the past week reported a daily average of at least one-half serving of fruit and one-half serving of vegetables more than adolescents who reported never helping with dinner. Dinner preparation frequency was also inversely associated with soft drink consumption among female adolescents and fried food consumption among male adolescents with all results reported as statistically significant (p < 0.01) [12]. In Canadian children aged 10–11 years, a cross-sectional study found that involvement in home food preparation was associated with a one serve increase in fruit and vegetable intake (p < 0.001) and higher self-efficacy for selecting and eating healthy foods, in comparison to children who were not involved [9]. In addition, previous cooking experiments and workshops have shown positive effects on liking, willingness to taste and intake immediately after the cooking session [10, 46–48]. The current study tried to explore these effects over a longer period of time in a more real-life setting in the home environment without the potential attractiveness of a workshop in a more interesting new setting.

Family-based interventions are increasingly being conducted using digital tools and have shown positive results for improving healthy cooking behavior. For example, a virtual cooking intervention designed for children aged between 8 and 18 years, was conducted among 60 children and 43 families [49]. Children and their families followed virtual instructions over 5-consecutive weeks and learnt about different cooking methods, importance of eating fresh and nutrient dense foods and the benefits of family meals. The results showed that the intervention improved children’s’ cooking self-efficacy (p < 0.001), nutrition knowledge (p = 0.005), and self-efficacy for consuming fruits and vegetables (p = 0.02) [49]. This study portrays the potential of apps that promote cooking within the family environment but did not measure potential effects on intake. Although the current feasibility study showed limited effect on dietary behavior, it is recommended that digital interventions with more resources available are developed and tested. The home environment may be more effective than the school environment for improving dietary behavior [3] and thus evidence that builds upon our study is warranted. These digital intervention methods could also be a good opportunity to improve the participation of parents in a school-based intervention bringing the two most important food environments stronger together.

As mentioned before, also in other studies no results on dietary intake were measured or reported. One reason for this is the difficulty of dietary intake assessments. Digital solutions with automated food recognition did not yet solve this issue and these tools are often not validated [50]. In the current study, some participants expressed their concerns in relation to difficulties in weighing the food or mentioned that they forgot to capture pictures before or after the meal was consumed. AI-image based mobile applications which include automatic food recognition and food volume estimation could be a next development step to decrease the participant and researcher burden of weighing and evaluating the foods [51] designed and developed my multidisciplinary teams that also involve the end-users [25]. According to a global survey, emphasis should be given to validated, user-friendly, free of charge apps that incorporate local food databases [52]. Furthermore, based on information on normal meal eating schedule, the app could send reminders to notify the user to capture image(s) before and after meal consumption.

Moreover, in future studies, if siblings are participating, they could also be included in the preparation procedure to avoid discomfort and imbalance between the family members. Additionally, more education on how to participate in the study might be needed to set clear goals and give parents easier directions on how to explain the procedure to their children.

### Strengths and limitations

The strength of this study is that it was conducted under real-life home settings. This can provide researchers with a realistic idea of the challenges and advantages of conducting such a trial, allowing larger and more effective digital interventions to be developed and evaluated.

A key limitation of this study was that it was conducted during the height of COVID-19. This situation may have led to the small non-representative sample size which required shortening the duration of the intervention period (from 12 to 10 weeks by removing two recipes). Furthermore, recruitment efforts had to take place online instead of in-person (e.g., through schools), making it difficult to reach enough and a broad range of participants. Another recruitment wave for more participants could not be achieved, as the project funds were only available for IT support for one year, therefore, no funds were available for the app to be managed beyond the timeframe of this study. Moreover, the study was limited to Swiss residents, and may have been only interesting to those already motivated to make healthy changes for their families, as the requirement was conducted via social media, therefore, the results cannot be generalized to the whole population. The inclusion and exclusion criteria may have been too strict and resulted in low participant uptake. In hindsight, it may have been beneficial to increase the age range of included children and allow those who liked the target items to participate, while considering these children in the analysis. Provided after the participants had done their grocery shop for the week, adding a hurdle to participation which might have been even more so due to the COVID-19 situation that stimulated a lower shopping frequency. The planning of the intervention meals should therefore be more flexible i.e., frequent deliveries of the week’s recipe, and take the shopping time into account. Furthermore, it should be acknowledged that the convenience sample of 40 parents who were asked their opinion of ‘unpopular’ food among their children were a non-representative sample and in the future, more validated research should be sought to choose target food items. In addition, whole-meal pasta is not a product that is disliked by many children and might get easily accepted, which might have impacted the results. It is also likely that mainly parents believe that children show lower acceptance of whole grain products or that they don’t like the taste themselves [35]. It is not known whether the children in the study disliked any other ingredients in the provided recipes, which may be a reason why some children did not eat their meals. Regarding recipe characteristics, some participants and children perceived the quantity of the study target foods (i.e., Brussels sprouts) during the study period as large. Some other family members did not appreciate the recipes and wished to quit the participation. Therefore, some flexibility in the proposed recipe ingredients as well as differentiation in terms of portions is highly important in future interventions. Last, but not least, the whole project was set-up and implemented during the COVID-19 pandemic in 2020 which influenced the recruitment of participants as we had to find solutions via social-media. It also made the intervention more difficult for parents. At the time of the intervention, the governmental advice was to limit grocery shopping to a minimum. Because the intervention recipes were only made available once a week, this was more complicated for parents to implement and schedule. However, we also see that this procedure shows potential for the future as digital interventions will enable a direct, personalized approach in a home-setting.

This feasibility study has given some insight into how to improve future family cooking app development. Apps can be further developed to reach parents and children of various age groups and spoken languages. Ideally, several recipes could be provided and personalized to target different types of food items, with different ingredient combinations, different ways of food preparation and ways of serving it. It could also provide educational aspects, such as basic nutritional information for each food item to build the knowledge on specific nutrients and health benefits adapted to child-friendly interface. The recipes could also be based on food preferences and a variety of skills, to involve children in cooking with different gamification techniques aimed at stimulating healthy eating patterns. Lastly, recruitment efforts should be improved as studies with larger samples are needed to be able to draw conclusions on behavior change, although no studies examine to what extent real-life eHealth interventions have on large populations [30].

## Conclusion

The study aimed to test the feasibility and impact of an innovative pilot intervention for a home-based cooking program, using a smartphone application. Results revealed no significant differences between intervention groups or at different intervention time points. However, a slight increase of liking of the initially disliked whole-meal pasta was seen after the children were involved in cooking the intervention recipes, no changes were found for the liking of Brussels sprouts. This pilot study can be used as a base for future trials with larger samples focusing on improving the dietary behavior of children using digital tools. Innovative technology can play a key role in accelerating dietary behavior improvement, saving time for parents and engaging families to cooking and consuming healthier meals.

## Data Availability

The datasets generated during and/or analysed during the current study are available from the corresponding author on reasonable request.
